# Construct and clinical verification of a nurse-led rapid response systems and activation criteria

**DOI:** 10.1186/s12912-022-01087-7

**Published:** 2022-11-14

**Authors:** Yuchen Wu, Jiaming Wang, Fan Luo, Dan Li, Xue Ran, Xuanlin Ren, Lixiu Zhang, Jingyun Wei

**Affiliations:** 1 Lanzhou University First Affiliated Hospital, Chengguan District, Lanzhou, 730000 Gansu Province China; 2 Gansu University of Traditional Chinese Medicine, Lanzhou, 730000 China; 3 Gansu Provincial Hospital, Lanzhou, 730000 China

**Keywords:** Nurse-led, Rapid response system, Rapid response teams, Activation criteria, Success rate of rescue

## Abstract

**Background:**

Effective team leadership and good activation criteria can effectively initiate rapid response system (RRS) to reduce hospital mortality and improve quality of life. The first reaction time of nurses plays an important role in the rescue process. To construct a nurse-led (nurse-led RRS) and activation criteria and then to conduct a pragmatic evaluation of the nurse-led RRS.

**Methods:**

We used literature review and the Delphi method to construct a nurse-led RRS and activation criteria based on the theory of “rapid response system planning.” Then, we conducted a quasi-experimental study to verify the nurse-led RRS. The control group patients were admitted from August to October 2020 and performed traditional rescue procedures. The intervention group patients were admitted from August to October 2021 and implemented nurse-led RRS. The primary outcome was success rate of rescue.

**Setting:**

Emergency department, Gansu Province, China.

**Results:**

The nurse-led RRS and activation criteria include 4 level 1 indicators, 14 level 2 indicators, and 88 level 3 indicators. There were 203 patients who met the inclusion criteria to verify the nurse-led RRS. The results showed that success rate of rescue in intervention group (86.55%) was significantly higher than that in control group (66.5%), the rate of cardiac arrest in intervention group (33.61%) was significantly lower than that in control group (72.62%), the effective rescue time of intervention group (46.98 ± 12.01 min) was shorter than that of control group (58.67 ± 13.73 min), and the difference was statistically significant (*P* < 0.05). The rate of unplanned ICU admissions in intervention group (42.85%) was lower than that in control group (44.04%), but the difference was not statistically significant (*P* > 0.05).

**Conclusions:**

The nurse-led RRS and activation criteria can improve the success rate of rescue, reduce the rate of cardiac arrest, shorten the effective time of rescue, effectively improve the rescue efficiency of patients.

**Supplementary Information:**

The online version contains supplementary material available at 10.1186/s12912-022-01087-7.

## Introduction

The rapid response system (RRS) is a safety intervention for patients that facilitates direct consultation about a patient’s worsening condition from a team of specially trained individuals outside a critical care area [1]. The components of RRS include identification, response, feedback mechanism, and administration. The RRS is activated by a trigger mechanism composed of a single sign or multiple early warning signs to identify critical patients at potential risk. Activation is followed by interventions initiated by a response team (RRT) or medical emergency team (MET) to prevent risk events [2–5]. RRS have been used to provide a safety net for clinically deteriorating patients on floors/wards to decrease cardiac arrest rates and prevent unplanned transfers into the intensive care unit (ICU) [6, 7]. The activation criteria are the core part of the identification, which is used to guide the caller to activate RRS and to screen potentially critical patients in the hospital [2, 3, 6]. Early warning signs vary by institution and are composed of select physiological variables and predetermined parameters for each [6–8]. The RRT is the core part of the response, and an early intervention team is composed of physicians and nurses with certain rescue experience and professional skills, along with other designated personnel [9–11].

RRTs were first launched as a medical emergency team in Australia in the early 1990s. RRTs are now considered by some Western countries as an integral part of hospitals because of the cumulative effect of reducing hospital mortality and cardiac arrest [12–14]. The RRT is made up of multidisciplinary professionals such as critical care nurses, respiratory therapists, and physicians; nurses play a key role in RRT, and most RRS are led by physicians [11, 13, 15, 16]. Studies have reported positive results from nurse-led RRS; they can quickly activate RRS, reduce the hospital mortality, improve the clinical outcome, and reduce medical and social costs [9–11, 17, 18].

RRS provides early coordinated intervention to patients showing warning signs and prevents the occurrence of serious adverse events by quickly detecting patients whose condition is deteriorating and responding swiftly and effectively with a RRT [19]. Even though the RRS has been widely advocated and adopted, there is still evidence of ongoing suboptimal activation [2, 20]. The activation criteria of RRS are slightly different among medical institutions. RRS can be activated when a patient’s vital signs show serious abnormalities in some medical institutions. However, the activation criteria of RRS are a modified early warning system in others [2, 8, 16, 19–21]. Studies have shown no activation of the RRS after a patient met the criteria in 30 to 78% of cases and a median delay of 16 h in RRS activations from the time patient met the criteria [2, 6, 19]. Effective team leadership and good activation criteria can effectively initiate RRS to reduce hospital mortality and improve quality of life. Therefore, we developed a nurse-led RRS and activation criteria based on a literature review and the Delphi method and verified the pragmatic evaluation of nurse-led RRS and activation criteria.

## Materials and methods

### Research design

#### Phase 1

We developed a nurse-led RRS and activation criteria based on a literature review and the Delphi method. Information was extracted by systematically searching PubMed, EMbase, Web of Science, MEDLINE, China Biology Medicine Disc, (CBMdisc), China National Knowledge Infrastructure (CNKI), and the Wanfang database, followed by a preliminary construction of nurse-led RRS and activation criteria entry pool through panel discussions. The nurse-led RRS and activation criteria were then determined by the Delphi method. Delphi method is mainly sent questionnaire to experts by Wechat or email, and short message assisted reminder. According to the results of the consultation and the principle of information saturation, decide whether to carry out the next round of consultation. Indicators screening was based on the principle that the mean value of importance assignment ≥3.5 and the variable coefficient < 2.5.

#### Phase 2

**Fig. 1:**
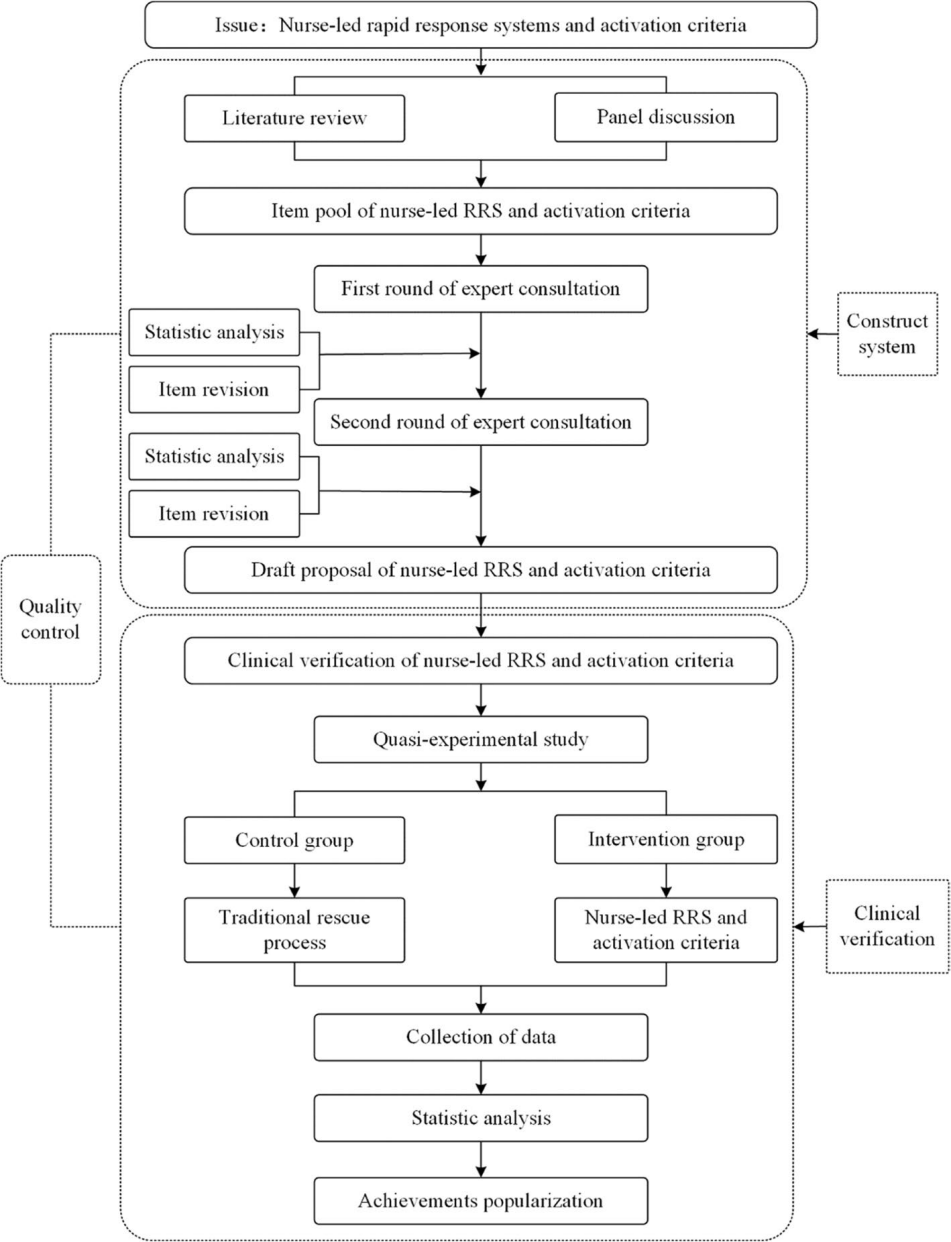
Study flowchart

We validated the pragmatic value of nurse-led RRS and activation criteria by a quasi-experimental study. The study flowchart was shown in the Fig. 1.

### Study population

#### Experts

The study included 20 experts from the critical care field in China who have been engaged in acute and critical care nursing, critical care education, critical care medicine, critical care management, and other related work for more than 10 years, with a senior title or above. Experts can continue to participate in the consultation and solution process of this study.

#### Patients

Patients were those who met the RRS activation criteria and volunteered to participate in the study and were hospitalized in the emergency department of a third Grade A hospital in Gansu Province, and the third Grade A hospital is a large urban hospital that experiences rapid response requirements frequently. The patients were admitted from August 2020 to October 2021. Eligibility patients are all meets the following criteria (1) Patients aged ≥18 years; (2) Patients who met the RRT activation criteria; (3) Patients or their legal guardian signed informed consent. We excluded patients less than 18 years old, who were pregnant, or who had CPR performed on admission.

### Sampling

The sample size of expert consultation is based on the information saturation principle, and we consulted 20 experts. Patients all came from a Grade A hospital in Lanzhou, Gansu Province. Sampling was by the convenience method. We included patients who met the inclusion criteria and were admitted to the emergency ICU from August to October 2020 and given the traditional rescue treatment and those who were admitted to the emergency ICU from August to October 2021 and treated with a nurse-led RRS.

### Ethical approval of the study protocol

The study protocol was approved (2020–215) by the Ethics Committee of Gansu Provincial Hospital (Lanzhou, China). Written informed consent was obtained from all study participants and their guardians.

### Intervention

The patients who met the inclusion criteria and were admitted to the emergency ICU from August to October 2020 were control group. The traditional rescue process, the emergency department physician responsibility system, was implemented in the control group. The nurses cooperated with the physicians to rescue patients according to the physician’s advice. The rescue process preliminarily judges the condition and provides life support at the same time; nurses assist in examination, invite specialist consultation according to the situation, and implement care according to the consultation opinion.

**Fig. 2:**
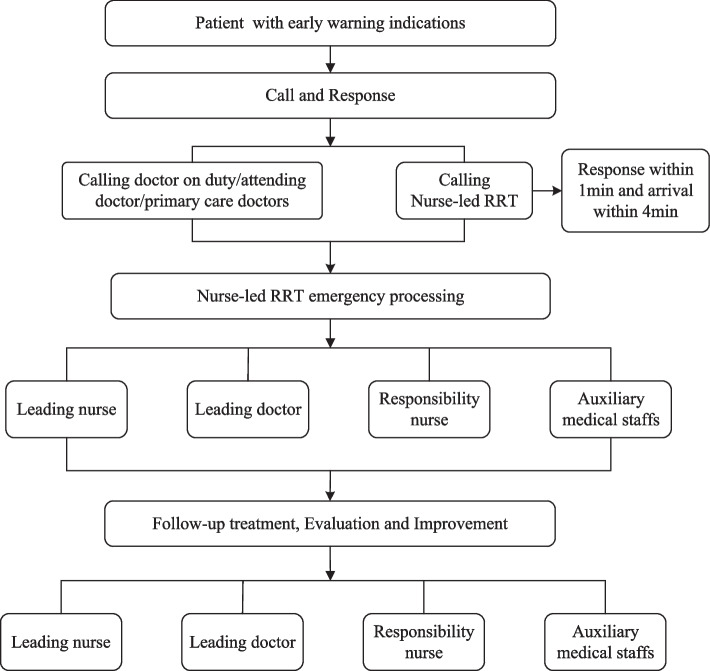
Nurse-led RRS implementation process

The intervention group strictly followed the nurse-led RRS implementation process (Fig. 2) and the RRS activation criteria which showed in the supplementary 2. Intervention group patients who were admitted to the emergency ICU from August to October 2021 and treated with a nurse-led RRS. Firstly, the nurse-led RRS implementation process was that everyone initiates the RRS according to the activation criteria, and the nurse-led RRT must respond within 1 min and perform rescue within 4 min. Secondly, the nursed-led RRT initiate emergency response. The leading nurse is responsible for evaluating the condition and directing the whole scene, the leading doctor is responsible for implementing rescue measures, others responsible for ECG monitor, defibrillation, establishing venous access, and so on. After the rescue, the nurse-led RRT will holding a consultation to discussion treatment and nursing care plan of the patient with attending physician. The detailed implementation process were showed in the supplement 1.

### Outcome indicators

Outcome indicators were the success rate of rescue, rate of cardiac arrest, unplanned ICU admission, and effective time of rescue.

(1) success rate of rescue(%) = Number of patients successfully rescued/ Total number of patients rescued*100%. The number of patients successfully rescued were included patients who survived at the time of discharge, and the total number of patients rescued were included patients.

(2) Rate of cardiac arrest(%) = Number of patients with cardiac arrests / Total number of patients rescued*100%.

(3) Unplanned ICU admission(%) = Number of emergency patients are admitted to ICU due to changes in their condition/ Total number of patients rescued*100%.

(4) Effective time of rescue: it was the time interval from cardiac arrest to stable recovery of vital signs.

### Statistical analysis

Variables are presented as either mean with standard deviation or median with an interquartile range (IQR), as appropriate. We compared the outcome index in implementing a nurse-led RRS and activation criteria and routine rescue process. Student’s t-test was used to compare continuous variables, and the chi squared test or Fisher’s test was used to compare categorical variables. All *P*-values were two-tailed, and P-values of less than 0.05 were considered statistically significant. IBM SPSS® Statistics (version 22.0; IBM Corp., Armonk, NY, USA) was used for all statistical analyses.

## Results

### Phase 1: developing nurse-led rapid response system and activation criteria

On the basis of the literature review and group discussion, an item pool of nurse-led RRS and activation criteria was developed that included 4 level 1 indicators, 14 level 2 indicators, and 88 level 3 indicators. There were 13 items that were revised, 7 items were merged, 2 items were deleted, and 5 items were added after the first round of expert consultation. After 2 weeks, we conducted the second round of expert consultation, and there were no items deleted and merged, but 8 items were revised. As a result, we preliminarily developed a nurse-led RRS and activation criteria that included 4 level 1 indicators, 14 level 2 indicators, and 88 level 3 indicators (Appendix 1).

There were 20 experts from the critical care field in China who were consulted and their characteristics are shown in Table 1; 20 questionnaires were issued and 20 were recovered with complete data in each round of expert consultation, so the response rate was 100%. The expert judgment coefficient (Ca) was 0.94, the familiarity coefficient (Cs) was 0.93, and the expert authority coefficient (Cr) was 0.935. Degree of expert consensus was tested by Kendall^’^s *W* and the results are shown in Table 2.

**Table 1:** Characteristics of experts

Items	Frequency	Proportion (%)
Age (years)		
30–40	5	25
40–50	11	55
>50	4	20
Education degree		
Undergraduate	7	35
Master	10	50
Doctor	3	15
Professional title		
Professor	6	25
Associate professor	10	55
Others	4	20
Working field		
Nursing management	5	25
Nursing education	3	15
Advanced practice nurse	9	45
Clinical medicine	3	15
Work seniority (years)		
10–20	6	30
20–30	9	45
>30	5	25

**Table 2:** Degree of coordination of expert consensus

Indicators	First round			Second round		
	Indicators number	Kendall’s *W*	P	Index number	Kendall’s *W*	P
Level 1 indicators	4	0.28	0.006	4	0.48	0.009
Level 2 indicators	14	0.16	0.000	14	0.12	0.030
Level 3 indicators	88	0.22	0.000	88	0.23	0.000

### Phase 2: pragmatic evaluation of a nurse-led RRS and activation criteria

This study included 203 patients, 84 in the control group and 119 in the intervention group. Analysis and comparison of the characteristics showed that the differences were not statistically significant (*P* > 0.05) between the two groups (Table 3).

**Table 3:** Characteristics of patients in two groups

Items		Control group (*n* = 84)	Intervention group (*n* = 119)	*t*/*χ*^2^	P
Age (years)		62.04 ± 16.51	57.85 ± 19.19	1.622	0.106
Sex	Female	26(30.95)	40(33.61)	0.159	0.690
	Male	58(69.05)	79(66.39)		
Department	Emergency medicine	49(58.33)	71(59.67)	1.365	0.503
	Emergency surgery	35(41.67)	48(40.34)		
Respiratory rate (bpm)		26.46 ± 12.97	24.43 ± 10.65	0.830	0.408
Heart rate (bpm)		101.57 ± 40.77	91.98 ± 43.54	1.500	0.135
Oxyhemoglobin saturation (%)		77.17 ± 24.58	72.66 ± 31.67	1.093	0.276
Systolic pressure (mmHg)		117.84 ± 54.39	112.03 ± 51.81	0.769	0.443
Diastolic pressure (mmHg)		70.18 ± 32.46	65.79 ± 27.24	1.044	0.298

### Outcome indicators between the two groups

The success rate of rescue in control group was 66.67% and that in intervention group was 86.55%, the difference in rate of rescue was significantly different (*P*<0.05).

The rate of cardiac arrest in the control group was 72.62%, and that in intervention group was 33.61%, which was significantly lower than that in control group (*P*<0.05). The rate of unplanned ICU admissions was 44.04% in the control group and 42.85% in the intervention group, but the difference between the two groups was not statistically significant (*P*>0.05) (Table 4). The average effective rescue time of the control group (58.67 ± 13.73 min) was shorter than that of the intervention group (46.98 ± 12.01 min), (*P*<0.05).

**Table 4:** Outcome indicators between the two groups

Items		Control (*n* = 84)	Intervention (*n* = 119)	Total (n)	*χ*^2^	P
Success rescue or not	Yes (n)	56(66.67)	103(86.55)	191	11.472	0.001
	No (n)	28(33.37)	16(13.45)	12		
Cardiac arrest	Yes (n)	61(72.62)	40(33.61)	101	29.968	0.000
	No (n)	23(27.38)	79(66.39)	102		
Unplanned ICU admission	Yes (n)	37(44.04)	51(42.85)	88	0.028	0.866
	No (n)	47(55.95)	68(57.14)	115		

## Discussion

### Nurse-led RRS and activation criteria can improve the success rate of rescue

Sudden cardiac arrest is a rapidly progressing event that affects millions of people every year. It is estimated that tens of thousands of people die from cardiac arrest every year, accounting for 15 to 20% of all deaths worldwide [22, 23]. However, only a very few patients can be successfully rescued. China’s national-wide cardiac arrest rescue success rate is only 1% ~ 5%, so improving the success rate of rescue is an urgent problem at present [5, 24]. This study found that the implementation of nurse-led RRS could improve the success rate of rescue from 66.67 to 86.55%, and the difference was statistically significant (*P*<0.05), which is consistent with the study of Ganju [24]. In traditional hospital rescue, nurses rely on physicians in emergency situations, carry out physicians’ orders, or even waste rescue time waiting for physicians’ orders [15–26]. In contrast, in nurse-led RRS, several rescue measures are carried out synchronously, and members have a tacit division of labor and cooperation in the process, therefore shortening the rescue time for patients and improving the success rate of rescue. On the other hand, the difference in the professional level and ability of physicians and nurses leads to different rescue effects [3, 13, 16]. Hence, the team members of nurse-led RRS were strictly selected and systematic training conducted, improving upon the RRT’s emergency response and collaboration capabilities.

### Nurse-led RRS and activation criteria can reduce the rate of cardiac arrest

The rate of cardiac arrest is one of important indicators to evaluate the effect of patient safety management. A survey study found that Eighty one hospitals had a dedicated cardiac arrest team in unite state [27]. In hospitals with mature RRS the cardiac arrest incidence is low and outcome better than traditionally believed. Before cardiac arrest is present in a significant number of patients and improved monitoring and earlier interventions may further improve outcomes by RRT [28]. Jones et al. confirmed that the RRT reduces the rate of cardiac arrest and reported that one cardiac arrest could be prevented in every 17 by RRT [29]. The study found the rate of cardiac arrest decreased from 72.62 to 39.01%, which consistent with others [14, 22, 23, 27, 28]. These findings may be related to the activation criteria that can be monitored in real time, which can guide callers in identifying high-risk patients early and activate the nurse-led RRS for intervention. In addition, the traditional rescue process does not have certain requirements for response and arrival time for rescue personnel, and the medical staff usually cannot arrive in time because they are dealing with other patients, thus delaying the rescue time. Therefore, the nurse-led RRT emergency process requires the RRT to respond within 1 min and arrive within 4 min [29] which reported by Cherry. et al.

### Nurse-led RRS and activation criteria can shorten the effective time of rescue

“Golden rescue time” emphasizes the importance of time in the rescue process of critical patients. If the treatment is not timely, there may be irreversible damage to the body. Studies have shown that there is a negative correlation between the on-site rescue time and the prognosis of patients, that is, the prognosis of patients is worse with the increase of on-site rescue time [30–32]. Nurse-led RRS shortened the effective rescue time from 58.67 min to 46.98 min, which is consistent with another study [30, 33]. Unclear division of labor and responsibilities in traditional rescue greatly affect the rescue time. First, nurse-led RRS and activation criteria provide an objective basis for the risk identification of critical patients that could guide RRT in quickly providing interventions according to the situation of patients. Second, the RRS has a clear division of labor, and the rescue process is standardized and orderly, which can avoid the existing problems such as rushing, repeated operation, ignoring major issues and so on. In short, nurse-led RRS obviously shorten the rescue time and improve the rescue outcome.

### Nurse-led RRS and activation criteria maybe affect the rate of unplanned ICU admission

Researchers have found that RRS can identify high-risk patients early and initiate timely intervention, transferring patients to a High Dependency Unit (HDU) according to their condition, which can reduce the work pressure on ICU staff and the economic burden of patients [31, 34, 35].

In this study, nurse-led RRS reduced the rate of unplanned ICU admission from 44.04 to 42.85%, but the difference was not statistically significant (*P* > 0.05). This may be related to the small sample size and physician decisions.

### Recommendation and limitation

Whether nurse-led RRS demonstrate benefit by improving patient safety and reducing the occurrence of adverse events remains controversial, but the nurse-led RRS makes the best use of human resources. Most existing RRS are activated by an early warning system (EWS) or modified EWS, and the activation criteria of nurse-led RRS are simple and feasible and include objective and subjective indexes. All hospital clinical staff and physicians should be educated about the purpose and process of Nurse-led RRS and RRT activation, this patient safety strategy is contributing to the ability of hospitals to rescue patients with signs of deterioration and reduce the occurrence of preventable adverse event. The limitation of the study was that we do not paly attention to the relevance of diagnosis on admittance, co-morbidties, APACHEII or SOFA score with nurse-led RRS.

## Conclusion

Compared with conventional traditional rescue procedures to an nurse-led RRS, nurse-led RRS and activation criteria can reduce the rate of cardiac arrest and unplanned ICU admissions. They can also shorten the rescue time for patients and improve the success rate of rescue. Most existing RRS are activated by an early warning system (EWS) or modified EWS, and the activation criteria of nurse-led RRS are simple and feasible and include objective and subjective indexes.

## Supplementary Information

**Additional file 1.**

**Additional file 2.**

## Data Availability

The datasets generated during and analyzed during the current study are not publicly available due to [REASON WHY DATA ARE NOT PUBLIC] but are available from the corresponding author on reasonable request. Data can be requested from the Ethics Committee (contact via the Ethics Committee of Gansu provincial Hospital, Lanzhou, Gansu, China, email: 751236201@qq.com) and the corresponding author (Email: 2569869312@qq.com) for researchers who meet the criteria for access to confidential data.
