# Supplementary material for: Construct and clinical verification of a nurse-led rapid response systems and activation criteria
Source: BMC Nurs. 2022 Nov 14;21:311. doi: 10.1186/s12912-022-01087-7 (PMC9661765; doi:10.1186/s12912-022-01087-7)
Supplement: Supplementary file 1 — Additional file 1. [file 12912_2022_1087_MOESM1_ESM.docx]

| **Level 1 Indicators** | **Level 2 Indicators** | **Level 3 Indicators** | |
| --- | --- | --- | --- |
| **Human Resource Management** | **Human Resource planning** | 1 Leading doctor | |
|  |  | 1 leading nurse, 1 responsibility nurse and 1 auxiliary nurse | |
|  |  | RRTs are formed in all important departments of the emergency, outpatient, medical and surgical systems | |
|  |  | Not involved in the region according to the principle of proximity to the division of responsibility RRT | |
|  | **Personnel qualification** | Leading doctor : Intermediate title and above or work experience ≥5 years | |
|  |  | Leading nurse: Intermediate title and above or work experience ≥10 years | |
|  |  | Responsible nurse: junior title and above or working experience ≥ 5 years | |
|  |  | Auxiliary nurse: junior title and above or working experience ≤ 3~5 years | |
|  |  | Professional knowledge and skills requirements | - Mastering the judgment and resuscitation techniques of cardiac arrest - Mastering resuscitation techniques of various types of shock - Mastering resuscitation techniques of dyspnea - Mastering resuscitation techniques of asphyxia - Mastering resuscitation techniques of acute chest pain - Mastering resuscitation techniques of severe cardiac arrhythmias - Mastering resuscitation techniques of persistent status epilepticus - Mastering resuscitation techniques of trauma - Mastering resuscitation techniques of ketoacidosis and hypoglycemic coma - Mastering resuscitation techniques of increased intracranial pressure |
|  |  | Clinical foresight | - Ability to identify potential or existing health problems in patients - Ability to identify signs of abnormal patient conditions |
|  |  | Strain ability | - Ability to respond effectively and timely to emergencies, implement crisis intervention and initiate emergency procedures when necessary - Ability to intervene with patients in the context of rapidly changing conditions in acute and critical care - Ability to identify the cause of cardiac arrest |
|  |  | Comprehensive analysis ability | - Ability to identify the cause of changes in a patient's condition - Ability to analyze the patient's condition from multiple perspectives |
|  |  | Judgmental evaluation ability | - Ability to correct errors based on theory and clinical experience - Ability to dynamic evaluation and documentation of resuscitation measures |
|  |  | Cognitive maturity | - Ability to make prudent and rational judgments |
|  |  | Management ability | - Ability to organize work in an organized manner during emergency resuscitation - Ability to organize team members to perform on-site first aid |
|  |  | Communication and coordination ability | - Ability to handle contentious, conflicting events - Ability to communicate effectively with patients, families, colleagues, and relevant departments - Ability to accurately present patient conditions and measures |
|  | **Personnel Training** | RRT member training content | - Theory and Practice of CPR - Team resuscitation process and cooperation - Single person CPR process - Theory and practice of trauma first aid techniques - Use of common resuscitation instruments - Application of common emergency drugs - First aid techniques such as suction and Heimlich maneuver |
|  |  | Training content for all medical staff in the hospital | - Activation criteria - Calling Process |
|  | **Personnel appraisal** | In-hospital medical staff activation standard familiarity assessment | |
|  |  | RRT regular basic theory, resuscitation techniques assessment | |
|  |  | Patient and family satisfaction assessment | |
|  |  | Satisfaction assessment of colleagues in the hospital | |
| **Environmental Information Management** | **Environmental Management** | To publish activation criteria in nursing units, wards, and crowded places in the hospital | |
|  |  | RRT members of each section wear the appropriate logo | |
|  |  | Resuscitation equipment | - Multifunctional defibrillator - Attractor - Blood gas analyzer - Tracheal Intubation Kits - Simple breathing capsule or portable ventilator - Oxygen bag or oxygen cylinder - Common emergency medications - Intravenous infusion tools - Transit Bed - Contact Equipment |
|  | **Information Management** | Summary analysis after each RRT resuscitation | |
|  |  | RRTs of various departments communicate with each other regularly | |
|  |  | Hospital-wide electronic patient record repository: Hospital-wide electronic medical records of admitted patients are available for searching | |
|  |  | Resuscitation patient electronic medical record library: all calls for patient resuscitation process and follow-up treatment record results | |
| **Quality Management** | **Structural quality** | Qualified rate of RRT healthcare configuration =100% | |
|  |  | RRT training assessment pass rate = 100% | |
|  |  | The rate of intactness of resuscitation equipment, items, drugs and contact tools = 100% | |
|  | **Process Quality** | In-hospital medical staff activation criteria familiarity ≥ 90% | |
|  |  | Effective call rate ≥ 80% | |
|  |  | Response rate within 1min = 100% | |
|  |  | Arrival rate within 4min = 100% | |
|  |  | Assessment accuracy rate ≥ 90% | |
|  |  | Resuscitation skills mastery rate = 100% | |
|  |  | Transit adverse event rate ≤ 3% | |
|  |  | Effective communication rate ≥ 90% | |
|  | **Result Quality** | Resuscitation success rate | |
|  |  | Unplanned ICU transfer rate | |
|  |  | In-hospital patient mortality | |
|  |  | Incidence of in-hospital patient cardiac arrest | |
|  |  | Effective time for resuscitation | |
| **Implementation Process** | **Step 1: Patient presents with early warning indications** | **Vital signs** | |
|  |  | Breathe | ＜8bpm or＞36bpm |
|  |  | Blood pressure | ＜70mmHg or＞220mmHg |
|  |  | Pulse | ＜40bpm or＞180bpm |
|  |  | Body temperature | ＞41℃ or＜32℃ |
|  |  | Blood oxygen saturation | Oxygenated state <80% |
|  |  | Shock index | (Pulse rate/Sbp) > 1.0-1.5 |
|  |  | **Respiratory System** | |
|  |  | Abnormal breathing | Respiratory arrest |
|  |  |  | dyspnea |
|  |  | apnea | Sudden interruption or cessation of hemoptysis, nervous or frightened expression, profuse sweating, scratching with both hands or indicating the throat |
|  |  |  | Extremely debilitating inability to cough, which may cause a sputum surge |
|  |  | **Circulation System** | |
|  |  | Peripheral circulation | Loss of aortic pulsation |
|  |  |  | Pale, clammy, cold skin and face, progressive drop in blood pressure |
|  |  |  | Superficial venous collapse, delayed capillary filling, and progressive drop in blood pressure |
|  |  | Abnormal electrocardiogram | ST segment elevation or downward shift or fish hook pattern |
|  |  |  | Inability to identify QRS wave groups or ST segments or T waves |
|  |  |  | Frequent ventricular anterior contractions (more than 5 per minute) |
|  |  | Chest Pain | Sudden onset of persistent chest pain of various nature (crushing, tearing, stabbing, burning, cutting) with radiating pain |
|  |  | **Nervous System** | |
|  |  | Consciousness disorders | Sudden coma |
|  |  | Pupillary abnormalities | Sudden loss of pupil reflex to light |
|  |  | Headaches | Severe headache, progressively worse |
|  |  |  | Restlessness and jet vomiting |
|  |  | Physical disorders | Sudden limb paralysis |
|  |  | Convulsive clonus | Bilateral tonic appearing clonus after falling to the ground |
|  |  |  | Tonic extension of extremities, torso inversion |
|  |  | **Other Systems** | |
|  |  | Digestive System | Massive vomiting of blood (blood loss >400mL in a short period of time) with black or bloody stools |
|  |  |  | Fluttering wing-like tremor with confusion and hallucinations |
|  |  |  | Pancreatic abdomen with signs of peritoneal irritation |
|  |  | Endocrine System | Deep and fast breathing with rotten apple smell, blood glucose value 33.3-66.6 mmol/L |
|  |  |  | Blood glucose <2.8 mmol/L with impaired consciousness |
|  |  |  | Thyroid crisis |
|  | **Step 2: Call and Response** | Calling patient duty/supervisor/primary care doctors | |
|  |  | Calling RRT | |
|  |  | RRT responds within 1 min and arrives within 4 min | |
|  | **Step 3: RRT emergency treatment** | Leading nurse: assess the condition, direct the whole scene, communicate | |
|  |  | Leading doctor: ensure airway is open, defibrillate or pace, compressions take over | |
|  |  | Responsibility nurse: compression cycle, ECG monitoring, assist in defibrillation | |
|  |  | Auxiliary nurse: establish access, administer catheterization, keep records | |
|  | **Step 4: Follow-up treatment** | Inpatient hospitalization | |
|  |  | Transfer to ICU / ward stay / surgery / automatic discharge / death | |
|  | **Step 5: Evaluation and Improvement** | Leading nurse: overall evaluation of the quality of resuscitation and making improvement plans | |
|  |  | Leading doctor: evaluation of resuscitation effects | |
|  |  | Responsibility nurse: evaluation of changes in condition | |
|  |  | Auxiliary nurse: evaluation of line condition and quality of records | |
