# Supplementary material for: Construct and clinical verification of a nurse-led rapid response systems and activation criteria
Source: BMC Nurs. 2022 Nov 14;21:311. doi: 10.1186/s12912-022-01087-7 (PMC9661765; doi:10.1186/s12912-022-01087-7)
Supplement: Supplementary file 2 — Additional file 2. [file 12912_2022_1087_MOESM2_ESM.docx]

**Activation criteria**

| **Vital signs** | Breathe | ＜8bpm or＞36bpm |
| --- | --- | --- |
|  | Blood pressure | ＜70mmHg or＞220mmHg |
|  | Pulse | ＜40bpm or＞180bpm |
|  | Body temperature | ＞41℃ or＜32℃ |
|  | Blood oxygen saturation | Oxygenated state <80% |
|  | Shock index | (Pulse rate/Sbp) > 1.0-1.5 |
| **Respiratory System** | Abnormal breathing | Respiratory arrest |
|  |  | dyspnea |
|  | apnea | Sudden interruption or cessation of hemoptysis, nervous or frightened expression, profuse sweating, scratching with both hands or indicating the throat |
|  |  | Extremely debilitating inability to cough, which may cause a sputum surge |
| **Circulation System** | Peripheral circulation | Loss of aortic pulsation |
|  |  | Pale, clammy, cold skin and face, progressive drop in blood pressure |
|  |  | Superficial venous collapse, delayed capillary filling, and progressive drop in blood pressure |
|  | Abnormal electrocardiogram | ST segment elevation or downward shift or fish hook pattern |
|  |  | Inability to identify QRS wave groups or ST segments or T waves |
|  |  | Frequent ventricular anterior contractions (more than 5 per minute) |
|  | Chest Pain | Sudden onset of persistent chest pain of various nature (crushing, tearing, stabbing, burning, cutting) with radiating pain |
| **Nervous System** | Consciousness disorders | Sudden coma |
|  | Pupillary abnormalities | Sudden loss of pupil reflex to light |
|  | Headaches | Severe headache, progressively worse |
|  |  | Restlessness and jet vomiting |
|  | Physical disorders | Sudden limb paralysis |
|  | Convulsive clonus | Bilateral tonic appearing clonus after falling to the ground |
|  |  | Tonic extension of extremities, torso inversion |
| **Other Systems** | Digestive System | Massive vomiting of blood (blood loss >400mL in a short period of time) with black or bloody stools |
|  |  | Fluttering wing-like tremor with confusion and hallucinations |
|  |  | Pancreatic abdomen with signs of peritoneal irritation |
|  | Endocrine System | Deep and fast breathing with rotten apple smell, blood glucose value 33.3-66.6 mmol/L |
|  |  | Blood glucose <2.8 mmol/L with impaired consciousness |
|  |  | Thyroid crisis |
